# Influence of Cervical Spine Mobility on the Focal and Postural Components of the Sit-to-Stand Task

**DOI:** 10.3389/fnhum.2017.00129

**Published:** 2017-03-28

**Authors:** Alain Hamaoui, Caroline Alamini-Rodrigues

**Affiliations:** ^1^Laboratoire de Physiologie de la Posture et du Mouvement, Institut National UniversitaireChampollion, Albi, France; ^2^Laboratoire Mouvement, Equilibre, Performance, Santé (EA 4445), Université de Pau et des Pays de l’AdourTarbes, France

**Keywords:** sit-to-stand, postural adjustments, focal movement, posturo-kinetic capacity, cervical spine, mobility

## Abstract

The aim of this study was to determine the influence of cervical spine mobility on the focal and postural components of the sit-to-stand transition, which represent the preparatory and execution phases of the task, respectively. Sixteen asymptomatic female participants (22 ± 3 years, 163 ± 0,06 cm, 57,5 ± 5 kg), free of any neurological or musculoskeletal disorders, performed six trials of the sit-to-stand task at maximum speed, in four experimental conditions varying the mobility of the cervical spine by means of three different splints. A six-channel force plate, which collected the reaction forces and moments applied at its top surface, was used to calculate the center of pressure displacements along the anterior-posterior and medial-lateral axes. The local accelerations of the head, spine, and pelvis, were assessed by three pairs of accelerometers, oriented along the vertical and anterior-posterior axes. Restriction of cervical spine mobility resulted in an increased duration of the focal movement, associated with longer and larger postural adjustments. These results suggest that restricted cervical spine mobility impairs the posturo-kinetic capacity during the sit-to-stand task, leading to a lower motor performance and a reorganization of the anticipatory postural adjustments. In a clinical context, it might be assumed that preserving the articular free play of the cervical spine might be useful to favor STS performance and autonomy.

## Introduction

The transition from sitting to standing, commonly named “sit-to-stand (STS)” is a very common daily task. In a study performed on eight wives of physicians and one man, McLeod et al. (1975) found that the STS task is performed four times an hour from 7 am to 10 pm. These results were refined by Dall and Kerr (2010) with an extended study on 140 participants, reporting an average of 60 STS movements per day and 3 per hour.

The STS task implies a rapid transition from a stable seated posture that offers a large contact area between the body and the supporting surfaces, to a less stable standing posture associated with a shorter base of sustentation, a higher location center of gravity and an extended articulated chain to stabilize. The standing posture is frequently a starting point for gait initiation, which involves a complex biomechanical process (Brenière and Do, 1991) preceding the rhythmic pattern of gait. Therefore, the ability to perform the STS movement is instrumental to autonomy, and many studies have been designed to unveil the kinematics and kinetics underlying this self-perturbing task (Burdett et al., 1985; Nuzik et al., 1986; Kralj et al., 1990; Schenkman et al., 1990; Riley et al., 1991; Coghlin and McFadyen, 1994; Roebroeck et al., 1994).

Most authors have divided the STS into two (Nuzik et al., 1986; Rodrigues-de-Paula Goulart and Valls-Solé, 1999), three (Schenkman et al., 1990; Coghlin and McFadyen, 1994; Roebroeck et al., 1994), or four (Kralj et al., 1990) different phases based on kinematics and kinetics data.

When considering the STS within the frame of the posturo-kinetic capacity concept (Bouisset and Zattara, 1983), a distinction should be made between a preparation phase, which corresponds to the anticipatory postural adjustments (APAs), and an execution phase during which the focal movement (FM) is performed. APAs allow for a better performance through a compensation of the disturbing forces associated with the FM (Bouisset and Zattara, 1981) or the generation of propulsive forces (Brenière and Do, 1991), and require postural chain mobility (Bouisset and Le Bozec, 1999). Lower articular free play of the lumbar pelvic area was associated with a reduction of the motor performance in several paradigms such as the pointing task (Lino and Bouisset, 1994), the pushing ramp effort (Le Bozec and Bouisset, 2004) or the STS task (Diakhaté et al., 2013).

However, there is a paucity of information regarding the role of the cervical spine, especially regarding the STS movement. In many kinematic studies of STS, total body movement is represented by a three-segment linkage (trunk-thigh-shank) pivoting about the foot (Shepherd and Gentile, 1994), and the influence of cervical spine mobility is taken into account only in a few experiments. In an earlier study, Jones and Hanson (1970) presented the trajectories of the head during the STS movement, traced from successive frames of motion picture. They constructed triangles from markers placed over the sternum, the seventh cervical vertebra and the head, exhibiting a cervical flexion during the first half of the trajectory. In a video-based kinematic analysis of the STS task, Nuzik et al. (1986) reported an average neck flexion of 8° during the first 35% of the movement cycle, followed by an extension phase. In an electromyographic study of the STS task, Rodrigues-de-Paula Goulart and Valls-Solé (1999) reported an activity of neck motor muscles (trapezius, sternocleidomastoideus) before and after the seat-off instant. Taken all together, these findings lead us to hypothesize that cervical mobility is involved in the preparatory and in the execution phases of the STS movement, and that a loss of cervical mobility is likely to impair both of them. In a neurophysiological point of view, it must also be noted that the cervical spine controls the orientation of the head, which is a frame of reference for action since it contains the visual and the vestibular systems (Pozzo et al., 1990). The main goal of this study was to assess the effect of cervical mobility restriction on the APAs and on the FM for the STS task. To this aim, biomechanical parameters were analyzed in different conditions varying cervical spine range of motion by means of three different collars.

## Materials and Methods

### Participants

Thirteen asymptomatic female participants (age: 23 ± 3 years; weight: 56 ± 9 kg; height: 163 ± 0,05 cm, BMI: 21 ± 3 kg/m^2^), free of any neurological or musculoskeletal disease participated in this study. Only female participants were included in this study to avoid any variation of cervical spine mobility that might be related to gender differences.

This study was carried out in accordance with the recommendations of the local “Ethics Committee for Movement Analysis.” All subjects gave written informed consent in accordance with the Declaration of Helsinki.

### Experimental Set-Up

#### Force Plate and Stool

A six-channel force plate (Bertec Corp., ref. 6012-15, Columbus, OH, USA), which collected the ground-reaction forces and moments applied at its top surface was used to calculate the coordinates of the center of pressure (CP) along the anterior-posterior axis (Xp), with the following formula: Xp = My/Rz (My is the moment around the medial-lateral axis and Rz is the vertical ground-reaction force).

**FIGURE 1:**
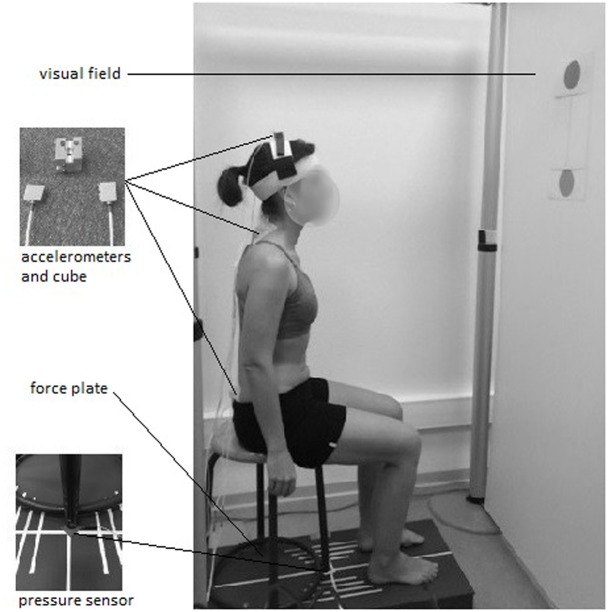
**Experimental set-up with force plate, accelerometers, pressure sensor, and visual field**.

A stool (height = 48 cm; depth = 39 cm) with four legs and a round wooden top (diameter = 30 cm) was screwed on to the force plate and used for the experiments (**Figure 1**).

To keep constant the friction forces between the stool top and the surface contact of the body, all participants wore the same kind of shorts.

#### Accelerometers

Three pairs of mono-axial accelerometers (FGP sensors, ref XA1010-B, ± 10g, Les Clayes Sous Bois, France), were used to assess the local accelerations of the pelvis, trunk, and head. Each pair was screwed on to a customized cube (length = 2 cm) with the two active axes located along the anterior-posterior and the vertical axes. The two first cubes were adhered to the skin with double-sided tape, at the level of the first sacral vertebra and first thoracic vertebra. The third cube was placed on top of the head using a system of Velcro bands (**Figure 1**).

#### Visual Field

The subject’s visual field consisted of a frontal white board (72 cm apart from the stool), on which two black disks (diameter = 10 cm) joined by a vertical line were placed at the subject’s eye level in the seated (lower disk) and standing (upper disk) postures (**Figure 1**). The participants were requested to focus their gaze on the lower disk at the beginning of the trial, and to follow the vertical line during the ascending phase of the STS, until they reach the upper disk. This visual field was designed to avoid unwanted cervical mobility due to a fixed visual target while the body was ascending. The experimentation room was lit with artificial lighting to obtain constant brightness.

#### Pressure Sensor

A pressure sensor was placed under the left front leg of the stool (**Figure 1**) to determine the “seat-off” instant.

#### Data Acquisition System

Data for all the recording devices were collected at 200 Hz with a 16-bit A/D converter board (model CompactDAQ with 9215 modules, National Instruments, Austin, TX, USA), controlled by a custom code written with Labview software (National Instruments).

### Cervical Collar

**FIGURE 2:**
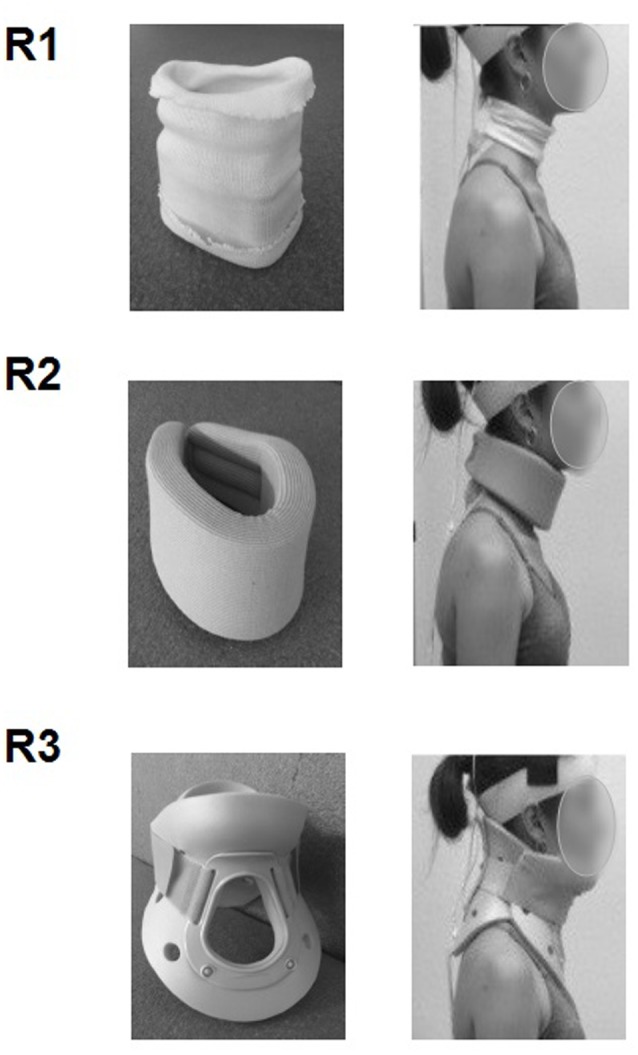
**Collars used to gradually restrict the mobility of the cervical spine: jersey tubular bandage (R1), foam cervical collar (R2), Philadelphia collar (R3)**.

Three different cervical collars (**Figure 2**) were used to vary the mobility of the cervical spine passively, in accordance with the study of Hartman et al. (1975), who measured cervical spine range of motion in flexion-extension, side bending, and rotation while wearing five commonly used cervical orthoses:

- Jersey tubular bandage (Neuss, Germany): minor restriction of cervical mobility.
- Foam cervical collar (Cooper, Melun, France): medium restriction of cervical mobility.
- Philadelphia collar (Variteks, Istanbul, Turkey): major restriction of cervical mobility.

### Procedure

The participants sat on the stool fixed on the platform with their upper limbs relaxed along their trunk, hips, and knees flexed to approximately 90°, barefoot and feet apart. Adhesive tape was put around the feet outline in order to keep the same positioning for every trial.

The subjects had to perform the “STS” task, which consisted in rising from the stool to reach the standing position as fast as possible, in response to a “Go” signal.

The STS was performed in four experimental conditions varying the mobility of the cervical area using the three above-mentioned collars: R0, no collar; R1, jersey tubular bandage; R2, foam cervical collar; R3, Philadelphia collar.

A training period was used to familiarize the subjects with the paradigm before recording.

Ten 3-s runs were performed in every condition, with a rest period of 30 s between runs and of 1 min between series.

The order of the experimental conditions was randomly assigned to avoid any order effect.

### Data Analysis

**FIGURE 3:**
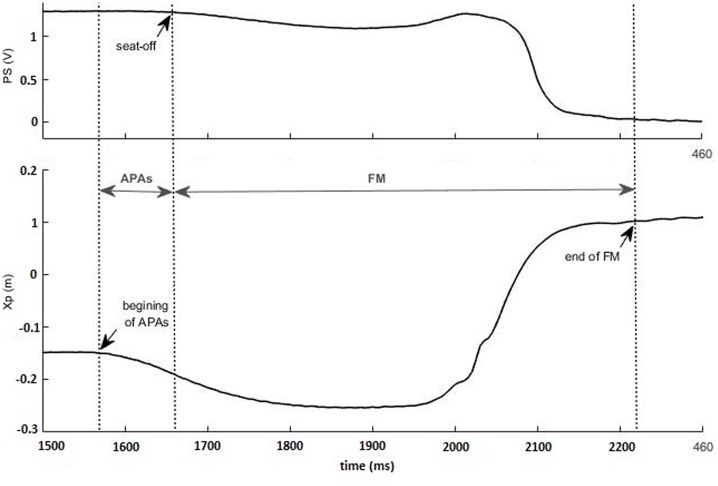
**Identification of the anticipatory postural adjustments (APAs) and of the focal movement (FM) phases, using pressure sensor (PS, in V) and anterior-posterior center of pressure (CP) (Xp, in m) traces: the beginning of the APAs and the end of the FM are identified in CP trace, whereas the seat-off is indicated in PS trace.** The recording was taken in a representative subject wearing a Philadelphia collar.

Three parameters were calculated to characterize the APAs and the FM of the STS task along the anterior-posterior axis. These two distinct phases of the task were considered to be separated by the seat-off instant, for which the curve of the pressure sensor begins its fall toward zero (**Figure 3**).

- Duration of anticipatory postural adjustments (*dAPAs*): delay between the instant of seat-off and the first inflection of the CP curve.
- Amplitude of APAs (*ΔXp*): difference between the maximum and the minimum CP values during the APAs phase.
- Duration of the focal movement (*dFM*): delay between the seat-off instant and the stabilization of the CP curve (beginning of the plateau region).

The different events (beginning of the APAs, seat-off, end of the FM) were based on the visual inspection of the curves, performed by a fully trained experimenter.

Data from the accelerometers were used to ensure that the head and trunk were kept still before the “Go” signal, and to discard trials in which the participants anticipated the instructions of the experimenter. The accelerometric signals were not used to separate the focal and the postural components of the task, because in complex movements such as the STS, some parts of the bony chain might be involved in both phases, with no clear distinction between the focal and the postural chains.

All parameters were calculated using a customized program written in MatLab software (The MathWorks, Inc., Natick, MA, USA).

A one-way repeated measures analysis of variance (ANOVA) was conducted for each dependent variable, with cervical mobility as a within-subjects factor. When statistical significance was reached, the ANOVA was followed by a within-subjects analysis of contrasts to compare the levels of the independent variable. As we presumed a significant difference between a reference condition (R0, no collar) and the three others ones (R1, R2, and R3), we used a simple contrast which compared R0 to each category (R1, R2, and R3). All statistical analysis were performed using the Statistical Package for Social Sciences (SPSS) software V22 (Chicago, IL, USA).

## Results

**FIGURE 4:**
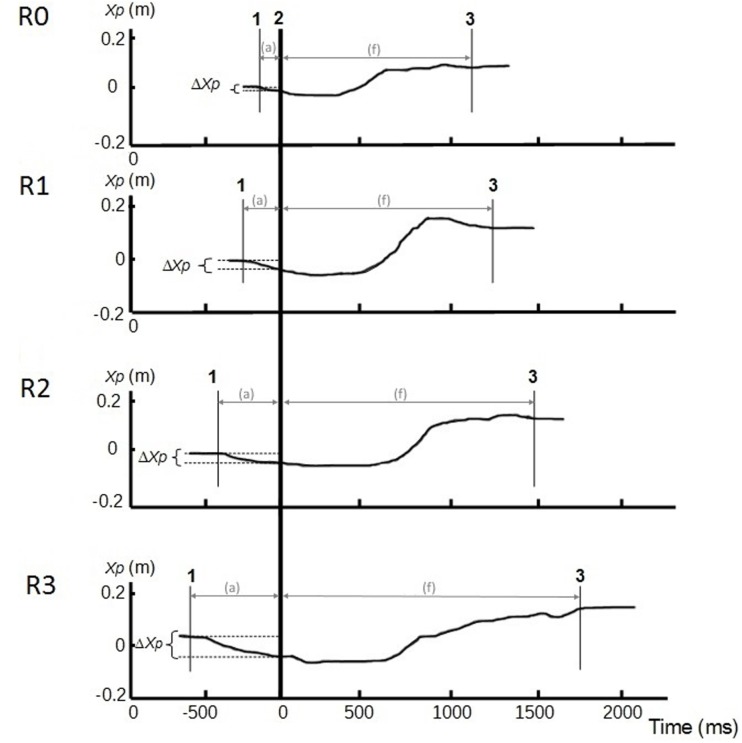
**Center of pressure traces along the anterior-posterior axis (Xp, in m) in different conditions of cervical spine mobility: no restriction (R0), jersey tubular bandage (R1), foam cervical collar (R2), Philadelphia collar (R3).** Recordings were taken in a representative subject. Line 1: CP onset; line 2: instant of “seat-off”; line 3: CP stabilization. APAs (a) phase is between lines 1 and 2, and focal phase (f) between lines 2 and 3. ΔXp represents the amplitude of the APAs.

**FIGURE 5:**
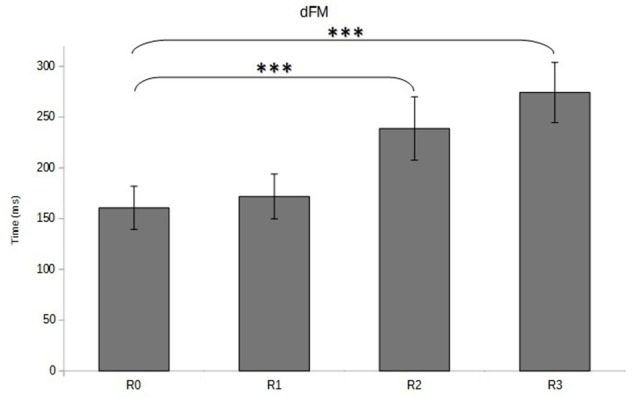
**Duration of focal movement (dFM, in ms) as a function of cervical spine mobility.** Means and standard deviations are presented in four different conditions varying cervical spine mobility using cervical orthoses: no restriction (R0), jersey tubular bandage (R1, minor restriction), foam cervical collar (R2, medium restriction), Philadelphia collar (R3, major restriction). ^∗∗∗^*p* < 0.001.

The visual inspection of CP traces along the anterior-posterior axis showed a gradual increase of APAs duration, APAs amplitude, and FM from R0 to R3 (**Figure 4**), which was confirmed by the statistical analysis. Indeed, the ANOVA revealed that the duration of the FM increased with the restriction of cervical spine mobility induced by the three collars (*p* < 0.001 for the overall effect) with significant variations in R2 and R3 relative to R0 (**Figure 5** and **Table 1**).

**Table 1:** Anticipatory postural adjustments (APAs) and focal mouvement (FM) parameters as a function of cervical spine mobility.

	*dAPA (ms)*	*Δ Xp (m)*	*dFM (ms)*
RO	14.41 ± 8.04	0.029 ± 0.012	195.3 ± 35.06
R1	15.76 ± 4.16	0.037 ± 0.019	191.42 ± 31.92
R2	22.38 ± 4.51	0.051 ± 0.023	241.66 ± 42.97
R3	27.77 ± 6.83	0.059 ± 0.022	268.29 ± 40.63
Overall effect	*p* < 0.01	*p* < 0.001	*p* < 0.001
p(R0/R1)	NS	NS	NS
p(R0/R2)	*p* < 0.05	*p* < 0.01	*p* < 0.001
p(R0/R3)	*p* < 0.001	*p* < 0.001	*p* < 0.001

*Mean ± SD of APAs duration (dAPAs), APAs amplitude (ΔXp), and focal movement duration (dMF) are presented in the different conditions varying cervical spine mobility: jersey tubular bandage (R1), foam cervical collar (R2), Philadelphia collar (R3).*

**FIGURE 6:**
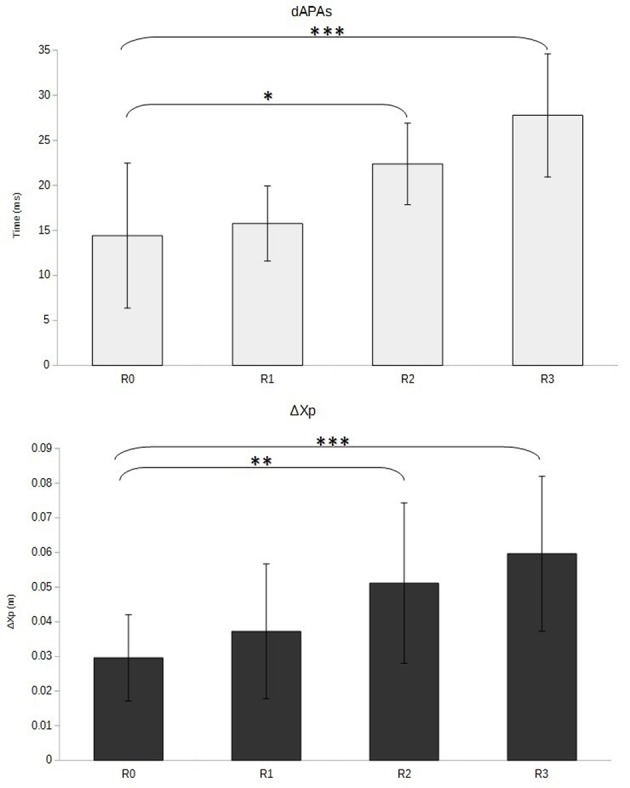
**Duration (dAPAs, in ms) and amplitude (ΔXp, in m) of anticipatory adjustments as a function of cervical spine mobility.** Means and standard deviations are presented in four different conditions varying cervical spine mobility using cervical orthoses: no restriction (R0), jersey tubular bandage (R1, minor restriction), foam cervical collar (R2, medium restriction), Philadelphia collar (R3, major restriction). ^∗^*p* < 0.05; ^∗∗^*p* < 0.01; ^∗∗∗^*p* < 0.001.

Similar variations were observed for the APAs, which presented higher duration (*p* < 0.01 for the overall effect) and magnitude (*p* < 0.001 for the overall effect) when cervical spine mobility was restricted (**Figure 5** and **Table 1**). The within-subjects contrast analysis showed significant differences between the R0 and R2 conditions (*p* < 0.05 for APAs duration and *p* < 0.01 for APAs amplitude) and between the R0 and R3 conditions (*p* < 0.001 for APAs duration and APAs amplitude) (**Figure 6**).

It must be noted that no significant variation was observed between R0 (no collar) and R1 (jersey tubular bandage) for any of the three analyzed parameters.

## Discussion

### Cervical Spine Mobility Is a Significant Parameter in the STS Task

The statistical analysis of the biomechanical parameters revealed that the FM duration of the STS task increased stepwise with the restriction of cervical spine mobility. As the subjects were requested to perform the task as fast as possible, it can be assumed that lower cervical spine mobility results in poorer performance for the STS task. This phenomenon could firstly be related to a lower ability to perform the FM when the articular free play of the cervical spine is restricted, in accordance with literature depicting the mobilization of this area during the focal phase of the STS. Indeed, Nuzik et al. (1986) reported a neck extension after the first 35% of the movement cycle, and Rodrigues-de-Paula Goulart and Valls-Solé (1999) showed that two motor muscles of the neck, namely trapezius and sternocleidomastoideus, present a noticeable EMG activity during the focal phase of the movement.

Secondly, the loss of cervical mobility may also impair the ability to perform efficient APAs, which represent a part of the central motor program that tends to reduce the early perturbations induced by the FM. In the paradigm of STS (Diakhaté et al., 2013) as in gait initiation (Brenière and Do, 1991), which include a shift of the center of mass (CM), APAs also contribute to the generation of the propulsive forces. It is assumed that APAs must be developed for the movement to be performed efficiently (Bouisset and Zattara, 1981). APAs involve postural chain mobility, and restricted articular free play has been shown to induce poorer performance in various paradigms, such as manual pointing or pushing (Bouisset and Le Bozec, 1999). The implication of cervical spine mobility during the anticipatory phase of the STS task is supported by existing literature, with Jones and Hanson (1970) representing a cervical flexion at the beginning of the STS trajectory in accordance with Nuzik et al. (1986). In the study by Rodrigues-de-Paula Goulart and Valls-Solé (1999), early EMG activity of the sternocleidomastoideus prior to the seat-off instant, very close to the onset of tibialis anterior that is the first muscle to be activated, has been reported. As the motor pattern of the APAs, including the motor muscles and the mobilized joints, is task specific (Bouisset and Zattara, 1981), the restriction of cervical spine mobility might not be easily compensated for. Consequently, APAs will become less efficient and require a significant increase of their amplitude and duration to counterbalance the disturbances associated with the FM, or to generate the propulsive forces. This adaptation of APAs programming is in line with a previous study exploring the effect of support base surface on the paradigm of shoulder flexion at maximum velocity while standing (Zattara and Bouisset, 1992). In this study, the authors showed that restricted support surface was associated with longer APAs and a lower performance of the FM (represented by the angular velocity peak).

### Cervical Spine as a Possible Guide for STS Kinematics

Beyond its key role as a part of the postural chain whose mobility reduces the disturbance associated with the FM in the STS, the cervical spine may also have other functions due to its upper location along the bony chain and articulation with the head. The early head flexion followed by an extension (Jones and Hanson, 1970), with a similar pattern for the trunk and pelvis (Nuzik et al., 1986), might provide the head and cervical spine with a guide function for STS kinematics. This function was suggested in a series of pictures taken in the burst mode in a few trials, with head mobility apparently starting and guiding the whole body trajectory. These hypotheses require an extended EMG and kinematic study to be assessed.

This specific role of the cervical spine could also be considered from a neurophysiological point of a view, as these vertebrae determines head orientation in space, and may vary visual field, vestibular activity, and cervical proprioception, which all provide substantial input for motor control (Paulus et al., 1984; Roll et al., 1989; Cullen, 2012). In the performance of complex tasks such as jumping, it has been shown that the head is sequentially stabilized in different positions, during several successive time periods (Pozzo et al., 1990). Hence, restricted articular free play in the cervical area might also impair the sensitive and sensory flow used by the CNS to control the STS task.

### APAs Are Adaptable

The analysis of CP displacements according to the seat-off instant showed that APAs duration and magnitude increased stepwise with the restriction of cervical spine mobility, suggesting that the local variations of the articular free play were integrated in the central programming of the task. This fine tuning of APAs is in line with literature demonstrating their adaptability to different biomechanical parameters, such as the size of the base of support (Yiou et al., 2007), or additional loads (Bouisset and Zattara, 1981). It may now be assumed that APAs are also adaptable to spine mobility variations.

### Clinical Implications

To date, little attention has been paid to cervical spine mobility in the STS task, either in a physiological perspective or in treatment and prevention strategies. It is well know that restriction of cervical spine mobility is frequent in the elderly (Lansade et al., 2009) or in neck pain syndroms (Cagnie et al., 2007), and results from this study suggest that it may impair the ability to perform the STS task. As a consequence, preserving or extending the cervical articular free play in these cases, using rehabilitation techniques or adapted physical programs, may be useful to preserve functional autonomy. Indeed, it must be recalled that the STS, which is considered a fundamental prerequisite for daily activities (Boukadida et al., 2015), is performed in mean 60 times per day (Dall and Kerr, 2010).

Elsewhere, the prescription of cervical collars, although they may represent an efficient tool to relieve pain or to favor cervical spine recovery (Muzin et al., 2007), should take into account their negative effect on the STS task, especially for patients presenting a loss of functional autonomy.

## Conclusion

This study has shown that passive restriction of cervical mobility results in a lower motor performance in the STS task, with an adaptation of the APAs, which become longer and larger. It is assumed that cervical spine mobility is an integral part of both the postural and the focal components of the STS task, with potential implications in rehabilitation strategies and adapted physical activity programs.

## Author Contributions

AH contributed with project creation, data analysis, and drafted the manuscript. CA-R contributed with project creation, data collection, and data analysis. AH and CA-R discussed the results and revised the manuscript.

## Conflict of Interest Statement

The authors declare that the research was conducted in the absence of any commercial or financial relationships that could be construed as a potential conflict of interest. The handling editor currently co-hosts a Research Topic with one of the authors AH, and confirms the absence of any other collaboration. He states that the process met the standards of a fair and objective review.
